# Dietary and Lifestyle Factors Associated with Dyspepsia among Pre-clinical Medical Students in Ajman, United Arab Emirates

**DOI:** 10.5195/cajgh.2016.192

**Published:** 2016-08-15

**Authors:** Noorallah Jaber, Marwa Oudah, Amer Kowatli, Jabir Jibril, Inbisat Baig, Elsheba Mathew, Aji Gopakumar, Jayakumary Muttappallymyalil

**Affiliations:** 1 Gulf Medical University, Ajman, United Arab Emirates;; 2 Department of Community Medicine, Gulf Medical University, Ajman, United Arab Emirates;; 3 Statistical Support Facility, Gulf Medical University, Ajman, United Arab Emirates

**Keywords:** dyspepsia, lifestyle factors, dietary factors, smoking, analgesic, alcohol, medical students

## Abstract

**Introduction:**

Dyspepsia is a common gastrointestinal diseases worldwide with a prevalence ranging from 7 to 40%. Dyspepsia, more commonly known as heartburn or indigestion, is defined as one or more of the following symptoms: postprandial fullness, early satiation (the inability to finish a normal size meal), or epigastric pain or burning for at least 3 months in the past year. Dyspepsia has been studied extensively, but little is known of factors associated with dyspepsia among medical students.

**Objectives:**

The purpose of this study was to analyze the prevalence of dyspepsia and to evaluate the association between lifestyle and dietary factors associated with dyspepsia among pre-clinical medical students in Ajman, United Arab Emirates.

**Methods:**

A cross-sectional survey study was conducted among pre-clinical medical students at Gulf Medical University, Ajman and collected basic demographic data, dyspepsia prevalence, dietary factors, and lifestyle factors. Data was analyzed using Microsoft Excel and SPSS software. Descriptive statistics were used to summarize the participant characteristics. Chi-square tests were used to test the association between dietary and lifestyle factors and dyspepsia. Logistic regression was used to measure the association of predictors (dietary and lifestyle factors) on the odds of having dyspepsia, independently. Multinomial logistic regression was used to examine the full association of predictors on the odds of having dyspepsia.

**Results:**

The resulting sample was 176 pre-clinical medical students, with a mean age of 20.67 ± 2.57 years. A total of 77 (43.8%) respondents reported having dyspepsia while 99 (56.2%) did not. There was a significant association between smoking and dyspepsia (*p*<0.05), as well as a marginally significant association between inadequate sleep and dyspepsia (p<0.10). There was no significant association with alcohol or analgesic use on dyspesia. Dietary habits showed no association with dyspepsia.

**Conclusion:**

Dyspepsia was reported by 43.8% of the repondents. These findings emphasize the importance of improving lifestyle and dietary factors associated with dyspepsia and raising awareness of reducing risk factors associated with dyspepsia. Further studies are needed on dyspepsia in a larger cohort of students in order to fully understand the complexity of this problem and be able to generalize the findings to other cohorts.

Dyspepsia is a term that is often used to characterize abdominal pain centered in the epigastrium and is often combined with other gastrointestinal disorders. Historically, ‘dyspepsia’ originates from the Greek ‘δυς-’ (dys-) and ‘πέψη’ (pepse), which means indigestion.^1^ In the mid-18th century, it was thought to be one of the ‘nervous disorders,’ along with hypochondria and hysteria.^2^ The Rome criteria was developed to classify the functional gastrointestinal disorders (FGIDs), disorders of the digestive system in which symptoms cannot be explained by the presence of structural or tissue abnormality, based on clinical symptoms. Rome II, defined dyspepsia as a pain or discomfort centered in the upper abdomen,^3^ specifically one or more of the following symptoms: postprandial fullness, early satiation (meaning inability to finish a normal size meal) epigastric pain or burning with at least a 3 month history in the last year.^4^ Some disorders may cause dyspepsia, such as gastroesophageal reflux disease (GERD), peptic ulcer, lactose intolerance, cholecystitis, anxiety or depression, stomach cancer, and often as a side effect of alcohol or medication.^5^ One of the risk factors for dyspepsia is lifestyle habits (e.g. smoking, increased caffeine intake) and non-steroidal anti-inflammatory drug use (e.g. aspirin), which is more relevant to ulcer dyspepsia.^6^

A study conducted in Columbia (N=937 students) on the prevalence of dyspepsia, concluded that around 40% of students had frequent dyspepsia.^7^ The annual incidence of dyspepsia was 9–10% and chronic dyspepsia was 15% with frequency of occurrence greater than 3 months in a year.^8^,^9^ Certain studies defined dyspepsia as “upper abdominal pain” in which prevalence of uninvestigated dyspepsia ranges from 7% to 34.2%.^10^–^15^ A study examining gastrointestinal symptoms in a multiracial Asian population reported that the prevalence of uninvestigated dyspepsia (a type of functional dyspepsia in which symptoms do not clearly fit standard definitions) is lowest in Singapore at 7.9% and highest in New Zealand with 34.2% of the population affected by the disease.^14^ Dyspepsia prevalence was reported as 7–8% in South East Asia,^14^ 30.4% in India,^15^ 14.5% in Scandinavian countries,^16^ and 23.0–25.8% in the U.S.^16^ The studies that defined dyspepsia as “upper gastrointestinal symptoms” reported prevalence of dyspepsia 23–25% with lower prevalence reported in Spain (23.9%).^17^ The prevalence rate of uninvestigated dyspepsia in US has been reported to be 32%, 38–41% reported in UK, and prevalence in Nigeria estimated at 45%.^18^–^23^

Dyspepsia has also been defined as pain centered in the upper abdomen and associated discomforts such as distention, belching, nausea or anorexia.^24^–^26^ A community-based study on the epidemiology of dyspepsia reported that 34.1% of respondents had dyspepsia within the past year.^27^ A study done in Kuwaiti dyspeptic patients reported that the overall prevalence of *Helicobacter pylori* (HP) infection related to dyspepsia was 88.5%.^28^ Various notable research studies have shown that dyspepsia is common, but the relationship between individualized symptoms, diet, and pathophysiology of dyspepsia remains unclear.^27^

Potential lifestyle factors associated with dyspepsia include tobacco, alcohol, and analgesic consumption. Furthermore, dietary habits that include consumption of smoked food, fast food, salty food, coffee/tea, and spicy food were associated with aggravating the symptoms of dyspepsia; while fruits, vegetables, and water were noted to improve the symptoms.^29^–^32^ Studies showed that smoking negatively affects dyspepsia symptoms by decreasing mucosal production, limiting the neutralizing base production, and decreasing blood flow to the inner layers of the stomach, interfering with normal physiological protective mechanisms of the stomach.^33^–^35^ The analgesic effects are explained as delayed gastric emptying, increased pyloric zonal contraction, and the release of acid.^36^ Alcohol has a similar role in damaging the stomach as analgesics, where it increases the production of acid.^37^

Though dyspepsia is a common problem among students, probably due to the interaction of various factors, there is no published research on the associations of dietary and lifestyle factors on dyspepsia among medical students. Previous studies have found risk factors such as diet, health, and lifestyle affect and exacerbate symptoms of dyspepsia. Therefore, we conducted a study among the pre-clinical medical students of one medical university in United Arab Emirates (UAE) to identify the magnitude of dyspepsia among them and the associated risk factors. This is one of the first of its kind in the UAE that has been designed to provide a better understanding of the problem, as well as to guide future lifestyle intervention programs.

## Methods

This cross-sectional survey study was conducted among pre-clinical medical students in Gulf Medical University, Ajman, UAE from September 2013 to March 2014. The total number of students in the three pre-clinical years of MBBS (MBBS year 1–3) was 176. All of the pre-clinical medical students who agreed to participate were included in the study.

As adequate TOEFL/IELTS score is a basic requirement for the university acceptance, all questionnaires were administered in English. A pilot feasibility study was conducted by administering the draft of the questionnaire to 5 students. After obtaining approval from the Ethics and Research Committees of GMU, the investigators recruited the participants using email and paper invitations. The survey was administered after obtaining the written consent of the participants. Data were collected in the classrooms during free time to ensure maximum participation. The investigators were present at the time of survey completion and were available to answer participant questions about the survey.

Self-report questionnaires were used to gather data on basic participant characteristics, dyspepsia prevalence, dietary factors, and lifestyle factors. The Short-Form Leeds Dyspepsia Questionnaire assessed the prevalence of dyspepsia.^18^ A separate questionnaire was used to examine lifestyle factors regarding smoking, consumption of alcohol, and use of analgesics. Here, analgesic use refers to over the counter analgesics such as paracetamol (acetaminophen), brufen (ibuprofen), aspirin, etc. A final questionnaire examined dietary factors such as the frequency of consumption of various food types. Short-Form Leeds Dyspepsia Questionnaire was scored as per the instructions in the manual. The summed up total score of the frequency and severity responses for each symptom was calculated. Categorized scores were calculated by rating the single most frequent or severe symptom from 0 (not at all) to 4 (once a day or more).The range was 0–32 and the cut off value for dyspepsia was 7 or more. A lifestyle questionnaire was made based on 2 sets of questions. The first set asked about the tobacco, alcohol, and analgesic intake. The next set assessed general lifestyle risk factors of dyspepsia and they were scored according to the number of risk factors marked as “often”; 1–3 risk factors as mild, 4–6 as moderate and 7–9 as severe. The sensitivity test of Short-Form Leeds Dyspepsia Questionnaire is 77.3% and the specificity is 73.2%.^38^

Data was analyzed using Microsoft Excel and Statistical Package for the Social Sciences (SPSS) software version 20 in four major steps. First, descriptive statistics were used to summarize the participant characteristics. Secondly, chi-square tests were used to test the association between dietary and lifestyle factors and dyspepsia. Thirdly, simple logistic regression was used to measure the association of predictors (dietary and lifestyle factors) on the odds of having dyspepsia, independently. Finally, multinomial logistic regression was used to examine the full association of predictors on the odds of having dyspepsia. A p<0.05 was considered as significant.

## Results

The mean age of the participants was 20.67±2.57 years, with 45.5% male participants. Table 1 shows the distribution of participant characteristics. Males did not differ significantly from females in terms of age distribution (20.90±2.83 years and 20.48±2.32 years, respectively). Participants aged 20 years and older made up 51.7% of the study population.

Table 2 summarizes lifestyle factors and dietary factors of the study participants. Tobacco users were defined as ever smokers and never smokers (who had not ever smoked in their life time). Ever smokers included both current and former smokers, as they smoked at least once in their life time, and the type of tobacco products considered were Cigar, Bidi, Shisha, and Midwakh (Dokha). Participants’ alcoholic habits were also categorized as ever alcohol consumer (including current and former users) and non-consumers. The majority of the participants were non-smokers (79%) and a higher proportion reported no alcohol consumption (86.4%). However, 97 (65.5%) reported having used analgesics often.

In terms of dietary consumption, majority of students were not consuming of smoked food, fast food, salty food, coffee/tea, and spicy food. Most of the participants (65.3%) reported that they were not performing any physical activities, and almost half (45.5%) of the participants self-reported inadequate sleep in the last two months.

Out of 176 participants, 77 (43.8%) participants reported experiencing dyspepsia and 99 (56.3%) reported no dyspepsia. Table 3 shows the association between the sociodemographic characteristics and dyspepsia. No statistically significant association was observed for age group, gender, nationality, and marital status with dyspepsia. With regard to batch of study, there was found statistical significant association with dyspepsia (p<0.05). Among the total participants with dyspepsia, 34 (44.2%) were belonged to the year 2 MBBS batch.

Table 4 shows the distribution of participants’ lifestyle factors, including smoking, alcohol, and analgesic usage in relation to dyspepsia. Among the respondents with dyspepsia, 22 (28.6%) were ever smokers whereas 55 (71.4%) were never smokers. The association observed was statistically significant (p<0.05). No statistically significant association was observed for alcohol consumption and analgesic use with dyspepsia.

Table 5 describes the distribution of participants according to dietary factors and their association with dyspepsia. Consumption of smoked food, fast food, and salty food has been associated with higher occurrence of dyspepsia. Among the participants with dyspepsia, 41 (53.2%) had history of inadequate sleep, which trended towards being significantly associated with dyspapesia (p<0.10). No statistical significant association was observed between for any other dietary factors and dyspepsia.

Chi-square test showed batch of study, participant’s tobacco use and inadequate sleep trended towards a significant association (p<0.10) with the occurrence of dyspepsia. In a secondary analysis, simple logistic regression was performed on these variables. All variables except inadequate sleep were statistically significant with p<0.05; however, inadequate sleep did show trends towards significance (p<0.10). In the final multinomial logistic regression model, tobacco use was found statistically significant (p<0.05). After adjusting for batch of study, it was concluded that tobacco use as the most predictable factor of dyspepsia (OR: 2.19, p<0.05, 95%CI: 1.02, 4.71). Moreover, both the factors are found to be independent without any confounding effect (since crude and adjusted odds ratios are almost same in both the variables). From Table 6, it was concluded that there is 2.2 greater odds (95%CI: 1.02, 4.71) of dyspepsia in tobacco users compared to non-users and 2.4 greater odds (95%CI: 1.16, 5.14) occurrence in Batch II MBBS students compared to other batches.

## Discussion

The study was conducted among 176 students of the junior three batches of the MBBS program offered at Gulf Medical University, Ajman, UAE to assess the prevalence of self-reported dyspepsia among pre-clinical students and its association with lifestyle and dietary factors.

In a study conducted by Novis et al., the population selected was healthy male and female students.^39^ The results found that out of 142 students 68 had developed dyspepsia during a period of 10 years.^39^ In a study conducted among randomly selected people in Peru, prevalence of dyspepsia was 37.6%. The prevalence of dyspepsia decreased as age increased. Ethnicity and dyspepsia were highly associated.^40^

A study was conducted by Rashed et al., to determine the incidence and significance of detection of *H. pylori* in an Arab population, observed that among 116 patients with dyspepsia 89% had *H. pylori*.^41^ It was suggested that *H. pylori* might be hyper-endemic among Arab patients with dyspepsia.^41^

A study survey of functional dyspepsia among the ethnic Malays in a primary care setting found results in which of the married subjects, females were more likely to have functional dyspepsia and psychosocial symptoms than men (6.3% vs. 1.9%).^42^ However, our study found that males were more likely to have dyspepsia in both married and single groups.

Our study found that smoking tobacco was associated with a significantly increased odds of having dyspepsia, similar to a study in Australia that identified smoking as an independent risk factor for dyspepsia (OR: 2.1, 95%CI: 1.3, 3.6).^43^ In our research 139 (78.9%) individuals who do not smoke have dyspepsia. A study conducted at University of Manchester in Saudi Arabia to determine the prevalence of smoking among medical and non-medical students showed that only 0.86% of students in college of medicine smoked tobacco products.^44^ A total of 74 (8.5%) reported smokers and 785 (91.5%) were nonsmokers. The same research highlighted the impact of alcohol on dyspepsia. It indicated that individuals who consume alcohol of 7 or more times per week have higher risk of reporting dyspepsia (OR: 2.3; 95% CI: 1.1, 5.0).^44^ In our study, 45.8% of participants who drank alcohol had dyspepsia compared to the participants who never drank alcohol (43.4%), while not significant these results warrant additional investigation.

Dyspepsia is more common in middle aged females.^45^ Published evidence suggested that independent risk factors for dyspepsia included the use of aspirin (OR: 2.2; 95%CI: 1.3, 3.7) and smoking (OR: 2.1; 95%CI: 1.3, 3.6), but not age, sex, marital status, educational level, income, or the use of alcohol, coffee, or nonsteroidal anti-inflammatory drugs.^44^ While there was a trend for increased dyspepsia among analgesics users, this relationship was not statistically siginifcant.

Results of a study in China indicated that prevalence of smoking was higher among college undergraduate students.^46^ A study conducted in Brazil shows that alcohol and tobacco were the substances more frequently used by the students, 85.2% and 16.3% respectively among medical students.^47^ It was also reported that 30 percent alcohol users in society were susceptible to developing dyspepsia.^48^ Another study reports alcohol consumption has been identified as one of the causes of dyspepsia.^49^

The management of uninvestigated dyspepsia traditionally included the reduction and cessation of coffee intake, although there was no strong research evidence that these changes in lifestyle could relieve the upper gastrointestinal symptoms.^50^ In our research, the percent of medical students that consumed coffee and tea on regular basis was 81 (46.0%). There was no significant association was found between coffee and tea intake and dyspepsia.

Nearly two-third of the medical students included in this study did not engage in physical activity. A similar research study conducted among medical students in the United Arab Emirates concluded that 77% of the students do not engage in any physical activity.^51^ In this study, engagement in physical activity was not associated with dyspepsia.

In summary, the following factors showed a trend in the relation with dyspepsia: tobacco use, alcohol use, and the use of analgesics. These factors were highly linked with the increasing prevalence of dyspepsia amongst pre-clinical students in Gulf Medical University, Ajman, UAE.

### Limitation

Findings of this study cannot be generalized to the general population of UAE or populations outside UAE; however, they may have important implications for student populations. Recall bias was present because some parts of the questionnaire required the students to recall previous events in the past few months. As most students in this study are expatriates, knowledge about family history may have been incomplete due to lack of knowledge about family members living elsewhere. Another limitation related to the concept of dyspepsia is that it was self-reported dyspepsia identified on the symptoms of indigestion, regurgitation, feeling of heartburn and nausea; the current study did not involve clinical confirmation of dyspepsia prevalence.

## Conclusion

Results of this study indicated that 43.8% of pre-clinical medical students had dyspepsia. History of smoking was significantly associated with dyspepsia while alcohol intake, analgesic use and other socio-demographic characteristics were not. Batch of study was also found association with dyspepsia as students’ stress and academic factors were related to their level of study. The dietary factors such as consumption of smoked food, fast food, salty food, fruits and vegetables, coffee, spicy food, water, and level of physical activity had no association with dyspepsia; however, inadequate sleep trended towards having a significant influence on dyspepsia. Although smoking habit and batch of study were found to be independent factors without any confounding effect, the present study concluded that tobacco use as the most predictive factor for dyspepsia among preclinical medical students in Ajman, UAE.

## Figures and Tables

**Table 1:** Participant descriptive characteristics

Variables	Groups	Gender	
		Male	Female
		N (%)	N (%)
Age group	≤20	36 (42.4)	49 (57.6)
	>20	44 (48.4)	47 (51.6)
Batch	2011	26 (49.1)	27 (50.9)
	2012	31 (51.7)	29 (48.3)
	2013	23 (36.5)	40 (63.5)
Nationality	Arabs	48 (55.8)	38 (44.2)
	Non- Arabs	32 (35.6)	58 (64.4)
Marital status	Married	3 (27.3)	8 (72.7)
	Single	77 (46.7)	88 (53.3)

**Table 2:** Distribution of participants based on lifestyle and dietary factors

Lifestyle factors	N (%)
Tobacco use	
Ever smoked	37 (21.0)
Never smoked	139 (79.0)
Alcohol consumption	
Ever drank alcohol	24 (13.6)
Never drank alcohol	152 (86.4)
Analgesics use	
Yes	97 (65.5)
No	51 (34.5)

Dietary factors	N (%)
Smoked food Consumption	
Yes	14 (8.0)
No	162 (92.0)
Fast food consumption	
Yes	59 (33.5)
No	117 (66.5)
Salty food consumption	
Yes	61 (34.7)
No	115 (65.3)
Fruit/Vegetable consumption	
Yes	74 (42.0)
No	102 (58.0)
Coffee/Tea consumption	
Yes	81 (46.0)
No	95 (54.0)
Spicy food consumption	
Yes	74 (42.0)
No	102 (58.0)
Beverage consumption during meals	
Yes	115 (65.3)
No	61 (34.7)
Physical activity	
Yes	61 (34.7)
No	115 (65.3)
Inadequate sleep	
Yes	80 (45.5)
No	96 (54.5)

***Note*. All dietary factors refer to dietary consumption. Details given for dietary consumption, physical activity and sleep are of the past two months.**

**Table 3:** Association between sociodemographic characteristics and dyspepsia

Sociodemographics	Groups	Dyspepsia		p value
		Yes	No	
		N (%)	N (%)	
Age group in years	≤20	38 (44.7)	47 (55.3)	0.875
	>20	39 (42.9)	52 (57.1)	
Gender	Male	36 (45.0)	44 (55.0)	0.76
	Female	41 (42.7)	55 (57.3)	
Batch	I MBBS	20 (37.7)	33 (62.3)	0.045
	II MBBS	34 (56.7)	26 (43.3)	
	III MBBS	23 (36.5)	40 (63.5)	
Nationality	Arabs	34 (39.5)	52 (60.5)	0.271
	Non-Arabs	43 (47.8)	47 (52.2)	
Marital status	Married	6 (54.5)	5 (45.5)	0.537
	Single	71 (43.0)	94 (57.0)	

**Table 4:** Association between substance use and dyspepsia

Lifestyle factors	Groups	Dyspepsia		p value
		Yes	No	
		N (%)	N (%)	
Tobacco use	Ever smoked	22 (59.5)	15 (40.5)	0.030
	Never smoked	55 (39.6)	84 (60.4)	
Alcohol use	Ever drank	11 (45.8)	13 (54.2)	0.825
	Never drank	66 (43.4)	86 (56.6)	
Analgesic use	Users	47 (48.5)	50 (51.5)	0.283
	Non-users	30 (38.0)	49 (62.0)	

**Table 5:** Association between dietary factors and dyspepsia

Dietary factors	Groups	Dyspepsia		p value
		Yes	No	
		N (%)	N (%)	
Smoked food consumption	Yes	7 (50.0)	7 (50.0)	0.623
	No	70 (43.2)	92 (56.8)	
Fast food consumption	Yes	29 (49.2)	30 (50.8)	0.305
	No	48 (41.0)	69 (59.0)	
Salty food consumption	Yes	29 (47.5)	32 (52.5)	0.460
	No	48 (41.7)	67 (58.3)	
Fruit/Vegetable consumption	Yes	30 (40.5)	44 (59.5)	0.465
	No	47 (46.1)	55 (53.9)	
Coffee/Tea consumption	Yes	35 (43.2)	46 (56.8)	0.894
	No	42 (44.2)	53 (55.8)	
Spicy food consumption	Yes	33 (44.6)	41 (55.4)	0.847
	No	44 (43.1)	58 (56.9)	
Beverage consumption during meals	Yes	53 (46.1)	62 (53.9)	0.391
	No	24 (39.3)	37 (60.7)	
Physical activity	Yes	28 (45.9)	33 (54.1)	0.675
	No	49 (42.6)	66 (57.4)	
Inadequate sleep	Yes	41 (51.2)	39 (48.8)	0.060
	No	36 (37.5)	60 (62.5)	

**Table 6:** Logistic regression of predictors of dyspepsia

Variables	Crude	Adjusted

	OR (95% CI)	OR (95% CI)
Study batch		
I MBBS	1.05 (0.50 – 2.25)	1.27 (0.58 – 2.78)
II MBBS	2.27 (1.10 – 4.69)*	2.44 (1.16 – 5.14)*
Ref: III MBBS	1 (--)	1 (--)
Tobacco use		
Ever smoked	2.24 (1.07 – 4.69)	2.19 (1.02 – 4.71)*
Ref: Never smoked	1 (--)	1 (--)

*Note*.

* Denotes p<0.05

## References

[1] Baron JH, Watson F, Sonnenberg A (2006). Three centuries of stomach symptoms in Scotland. Aliment Pharmacol Ther.

[2] Hare E (1991). The history of ‘nervous disorders’ from 1600 to 1840, and a comparison with modern views. Br J Psychiatry.

[3] Chey WD (2000). Accurate diagnosis of Helicobacter pylori. 14C-urea breath test. Gastroenterol Clin North Am.

[4] Tack J, Talley NJ, Camilleri M (2006). Functional gastroduodenal disorders. Gastroenterology.

[5] Harmon RC, Peura DA (2010). Evaluation and management of dyspepsia. Therap. Adv. Gastroenterol.

[6] Mahadeva S, Goh KL (2006). Epidemiology of functional dyspepsia: A global perspective. World J Gastroenterol.

[7] Caro JM, Ortiz SP, Melo CL (2008). Dyspepsia and reflux disease in adolescents. Rev Col Gastroenterol.

[8] Talley NJ, Weaver AL, Zinsmeister AR, Melton LJ (1992). Onset and disappearance of gastrointestinal symptoms and functional gastrointestinal disorders. Am J Epidemiol.

[9] Paré P (1999). Systematic approach toward the clinical diagnosis of functional dyspepsia. Can J Gastroenterol.

[10] Talley NJ, Zinsmeister AR, Schleck CD, Melton LJ (1992). Dyspepsia and dyspepsia subgroups: a population-based study. Gastroenterology.

[11] Talley NJ, Fett SL, Zinsmeister AR, Melton LJ (1994). Gastrointestinal tract symptoms and self-reported abuse: A population-based study. Gastroenterology.

[12] Agréus L, Talley NJ, Svärdsudd K, Tibblin G, Jones MP (2000). Identifying dyspepsia and irritable bowel syndrome: the value of pain or discomfort, and bowel habit descriptors. Scand J Gastroenterol.

[13] Bernersen B, Johnsen R, Straume B (1996). Non-ulcer dyspepsia and peptic ulcer: The distribution in a population and their relation to risk factors. Gut.

[14] Ho KY, Kang JY, Seow A (1998). Prevalence of gastrointestinal symptoms in a multiracial Asian population, with particular reference to reflux-type symptoms. Am J Gastroenterol.

[15] Shah SS, Bhatia SJ, Mistry FP (2001). Epidemiology of dyspepsia in the general population in Mumbai. Indian J Gastroenterol.

[16] Kay L, Jørgensen T (1994). Epidemiology of upper dyspepsia in a random population. Prevalence, incidence, natural history, and risk factors. Scand J Gastroenterol.

[17] Caballero-Plasencia AM, Sofos-Kontoyannis S, Valenzuela-Barranco M, Martín-Ruiz JL, Casado-Caballero FJ, López-Mañas JG (1999). Irritable bowel syndrome in patients with dyspepsia: a community-based study in southern Europe. Eur J Gastroenterol Hepatol.

[18] Shaib Y, El-Seraq HB (2004). The prevalence and risk factors of functional dyspepsia in a multiethnic population in the United States. Am J Gastroenterol.

[19] Jones RH, Lydeard SE, Hobbs FD (1990). Dyspepsia in England and Scotland. Gut.

[20] Jones R, Lydeard S (1989). Prevalence of symptoms of dyspepsia in the community. BMJ.

[21] Penston JG, Pounder RE (1996). A survey of dyspepsia in Great Britain. Aliment Pharmacol Ther.

[22] Moayyedi P, Forman D, Braunholtz D (2000). The proportion of upper gastrointestinal symptoms in the community associated with Helicobacter pylori, lifestyle factors, and nonsteroidal anti-inflammatory drugs. Leeds HELP Study Group. Am J Gastroenterol.

[23] Ihezue CH, Oluwole FS, Onuminya JE, Okoronkwo MO (1996). Dyspepsias among the highlanders of Nigeria: an epidemiological survey. Afr J Med Med Sci.

[24] Talley NJ, Colin-Jones D, Koch KL, Koch M, Nyren O, Stanghellini V (1991). Functional dyspepsia: A classification with guidelines for diagnosis and management. Gastroenterology.

[25] Holtmann G, Talley NJ (1993). Functional dyspepsia. Current treatment recommendations. Drugs.

[26] Armstrong D (1996). Helicobacter pylori infection and dyspepsia. Scand J Gastroenterol Suppl.

[27] Castillo EJ, Camilleri M, Locke GR (2004). A community-based, controlled study of the epidemiology and pathophysiology of dyspepsia. Clin Gastroenterol Hepatol.

[28] Abahussain EA, Hasan FA, Nicholls PJ (1998). Dyspepsia and Helicobacter pylori infection: Analysis of 200 Kuwaiti patients referred for endoscopy. Ann Saudi Med.

[29] Marsden K (1988). What doctors don’t tell you: Ulcers & indigestion - developing good gut sense. http://www.healthy.net/Health/Article/ULCERS_INDIGESTION/3357/4.

[30] Ganasegeran K, Al-Dubai SA, Qureshi AM, Al-abed AA, Am R, Aljunid SM (2012). Social and psychological factors affecting eating habits among university students in a Malaysian medical school: A cross-sectional study. Nutr J.

[31] Courteney H Indigestion help sheet. http://hazel-courteney.com/indigestion-help-sheet/.

[32] Akhondi-Meybodi M, Aghaei MA, Hashemian Z (2015). The role of diet in the management of non-ulcer dyspepsia. Middle East journal of digestive diseases.

[33] Abdulghani HM, AlKanhal AA, Mahmoud ES, Ponnamperuma GG, Alfaris EA (2011). Stress and its effects on medical students: A cross-sectional study at a college of medicine in Saudi Arabia. J Health Popul Nutr.

[34] Ramakrishnan K, Salinas RC (2007). Peptic ulcer disease. Am Fam Physician.

[35] Gralnek IM, Barkun AN, Bardou M (2008). Management of acute bleeding from a peptic ulcer. N Engl J Med.

[36] Talley NJ, Zinsmeister AR, Schleck CD, Melton LJ (1994). Smoking, alcohol, and analgesics in dyspepsia and among dyspepsia subgroups: Lack of an association in a community. Gut.

[37] Choices NHS (2014). Heartburn and gastro-oesophageal reflux disease. http://www.nhs.uk/Conditions/Gastroesophageal-reflux-disease/Pages/Introduction.aspx.

[38] Fraser A, Delaney BC, Ford AC, Qume M, Moayyedi P (2007). The Short-Form Leeds Dyspepsia Questionnaire validation study. Aliment Pharmacol Ther.

[39] Novis BH, Marks IN, Bank S, Sloan AW (1973). The relation between gastric acid secretion and body habitus, blood groups, smoking, and the subsequent development of dyspepsia and duodenal ulcer. Gut.

[40] Curioso WH, Donaires Mendoza N, Bacilio Zerpa C, Ganoza Gallardo C, León Barúa R (2002). Prevalence and relation of dyspepsia to irritable bowel syndrome in a native community of the Peruvian jungle. Rev Gastroenterol Peru.

[41] Rashed RS, Ayoola EA, Mofleh IA, Chowdhury MN, Mahmood K, Faleh FZ (1992). Helicobacter pylori and dyspepsia in an Arab population. Trop Geogr Med.

[42] Lee YY, Wahab N, Mustaffa N (2013). A Rome III survey of functional dyspepsia among the ethnic Malays in a primary care setting. BMC Gastroenterol.

[43] Nandurkar S, Talley NJ, Xia H, Mitchell H, Hazel S, Jones M (1998). Dyspepsia in the community is linked to smoking and aspirin use but not to Helicobacter pylori infection. Arch Intern Med.

[44] Abdulghani HM, Alrowais NA, Alhaqwi AI (2013). Cigarette smoking among female students in five medical and nonmedical colleges. Int J Gen Med.

[45] Carbone F, Holvoet L, Tack J (2015). Rome III functional dyspepsia subdivision in PDS and EPS: recognizing postprandial symptoms reduces overlap. Neurogastroenterol. Motil.

[46] Zhu T, Feng B, Wong S, Choi W, Zhu SH (2004). A comparison of smoking behaviors among medical and other college students in China. Health Promot Int.

[47] Petroianu A, Reis DC, Cunha BD, Souza DM (2010). Prevalence of alcohol, tobacco and psychotropic drug use among medical students at the Universidade Federal de Minas Gerais. Rev Assoc Med Bras.

[48] Hyams JS, Burke G, Davis PM, Rzepski B, Andrulonis PA (1996). Abdominal pain and irritable bowel syndrome in adolescents: A community-based study. J Pediatr.

[49] Colledge NR, Walker BR, Ralston SH (2010). Davidson’s principles and practice of medicine.

[50] Khot A, Polmear A (2011). Practical general practice: Guidelines for effective clinical management.

[51] Carter AO, Elzubeir M, Abdulrazzaq YM, Revel AD, Townsend A (2003). Health and lifestyle needs assessment of medical students in the United Arab Emirates. Med Teach.

